# Agua Salud Alphavirus Infection, Dissemination and Transmission in *Aedes aegypti* Mosquitoes

**DOI:** 10.3390/v15051113

**Published:** 2023-05-03

**Authors:** Swati V. Jagtap, Jorn Brink, Svea C. Frank, Marlis Badusche, Mayke Leggewie, Vattipally B. Sreenu, Janina Fuss, Esther Schnettler, Mine Altinli

**Affiliations:** 1 Bernhard-Nocht-Institute for Tropical Medicine, 20359 Hamburg, Germany; jagtap.swati@bnitm.de (S.V.J.);; 2 German Center for Infection Research, Partner Site Hamburg-Lübeck-Borstel-Riems, 20359 Hamburg, Germany; 3 MRC-University of Glasgow-Center for Virus Research, Glasgow G61 1QH, UK; 4 Institute of Clinical Molecular Biology (IKMB), Kiel University, 24105 Kiel, Germany; 5 Faculty of Mathematics, Informatics and Natural Sciences, University Hamburg, 20148 Hamburg, Germany

**Keywords:** Agua Salud alphavirus, insect-specific viruses, horizontal transmission, *Aedes aegypti*

## Abstract

Mosquitoes are competent vectors for many important arthropod-borne viruses (arboviruses). In addition to arboviruses, insect-specific viruses (ISV) have also been discovered in mosquitoes. ISVs are viruses that replicate in insect hosts but are unable to infect and replicate in vertebrates. They have been shown to interfere with arbovirus replication in some cases. Despite the increase in studies on ISV–arbovirus interactions, ISV interactions with their hosts and how they are maintained in nature are still not well understood. In the present study, we investigated the infection and dissemination of the Agua Salud alphavirus (ASALV) in the important mosquito vector *Aedes aegypti* through different infection routes (per oral infection, intrathoracic injection) and its transmission. We show here that ASALV infects the female *Ae. aegypti* and replicates when mosquitoes are infected intrathoracically or orally. ASALV disseminated to different tissues, including the midgut, salivary glands and ovaries. However, we observed a higher virus load in the brain than in the salivary glands and carcasses, suggesting a tropism towards brain tissues. Our results show that ASALV is transmitted horizontally during adult and larval stages, although we did not observe vertical transmission. Understanding ISV infection and dissemination dynamics in *Ae. aegypti* and their transmission routes could help the use of ISVs as an arbovirus control strategy in the future.

## 1. Introduction

Mosquitoes are common vectors of arboviruses, including medically important viruses such as Zika, chikungunya, dengue, and Yellow fever viruses. In addition to the arboviruses they transmit to vertebrates, an increasing number of insect-specific viruses (ISV) have been discovered in mosquitoes over the last decade [1,2,3]. ISVs are a diverse group of viruses that replicate in mosquitoes and mosquito-derived cell lines; however, they are unable to infect vertebrates and derived cell lines.

ISV studies have gained traction during the last decade as some ISVs can serve as vaccine platforms [4,5], and some can interfere with arbovirus infection [6,7,8,9,10]. ISV interference with arboviruses has been investigated mostly for flaviviruses [3] and has presented contradicting results ranging from interference to the facilitation of arbovirus infection [6,9,11,12,13]. However, there are still many open questions in terms of the interactions of ISV with their hosts, such as how they are maintained in nature and how they affect their mosquito hosts [13].

Despite the high number of insect-specific flaviviruses that have been discovered [14], to date, only five insect-specific alphaviruses have been found in mosquitoes, namely the Eilat virus (EILV) [15], Tai Forest alphavirus (TALV) [16], Mwinilunga alphavirus (MWAV) [17], Yada Yada Virus [18] and Agua Salud alphavirus (ASALV) [19]. This stark difference in their presence in natural populations compared to flaviviruses could be related to different transmission routes and interactions with their mosquito hosts. 

Alphaviruses encompass a large group of positive-sense single-stranded RNA viruses with a diverse host range, ranging from vertebrates (e.g., humans, monkeys, horses, birds, reptiles, and amphibians) to invertebrates (e.g., mosquitoes and ticks). Only two of the insect-specific alphaviruses have been isolated to date, namely ASALV and EILV. EILV was isolated from a pool of *Anopheles coustani* and has been shown to infect different mosquito species, including *Aedes albopictus*, *Ae. aegypti*, *Anopheles gambiae*, *Culex quinquefasciatus,* and *Cx. tarsalis* [15,20,21]. EILV can interfere with the replication of several arboviruses [22]; however, it is not yet known whether this is the case for other insect-specific alphaviruses, including ASALV. ASALV was isolated from *Culex declarator* mosquitoes and sampled in Panama [19], although the ASALV host range in vivo has not yet been investigated. In vitro, ASALV replicates both in *Ae. albopictus*-derived C6/36 and U4.4 cells [19] and *Ae. aegypti*-derived AF5 cells [23]. ASALV has been shown to interact with the major antiviral response in mosquitoes with RNA interference. RNA interference (RNAi) is a natural phenomenon that regulates gene expression and plays an important role in the regulation of viral infections in mosquitoes [24,25]. It can be divided into three distinct pathways in mosquitoes: the microRNA (miRNA), small interfering RNA (siRNA), and P-element-induced wimpy testis (PIWI)-interacting RNA (piRNA) pathway. The siRNA pathway is antiviral against all tested arboviruses [26] and ASALV, too [23]. The siRNA pathway is triggered by long double-stranded RNAs (dsRNA), for instance, replication intermediates, produced during viral replication. dsRNAs are then cut by Dicer2 into virus-derived siRNAs (vsiRNA) of 21 nucleotides (nt) in size, and vsiRNAs are loaded into the multi-protein, RNA-induced silencing complex (RISC). RISC can then target complementary viral RNA for subsequent cleavage, resulting in the inhibition of virus replication [27,28]. In addition to siRNAs, virus-derived piRNA (vpiRNA) of 25 to 29 nt in length have also been reported in infected mosquitoes and mosquito-derived cells [29,30]. In *Ae.aegypti*-derived cells, vpiRNA is produced through a ping-pong amplification cycle by Ago3 and Piwi5/6. The resultant vpiRNAs have a bias for either uridine at position one or adenine at position ten in the antisense and sense sequences, respectively (U1 and A10), and a complementary region of 10 nucleotides [29,30,31].

Here, we investigated ASALV infection and dissemination in *Ae. aegypti* through different infection routes (oral or intrathoracic injection) and its possible transmission routes. Understanding how ISVs interact with their mosquito hosts and transmission routes is important for the use of ISVs for arbovirus control tools in the future. 

## 2. Materials and Methods

### 2.1. Mosquitoes

The *Ae. aegypti* PAEA strain (obtained from Prof. Failloux, Institute Pasteur) was used in the experiments [32]. The *Cx. quinquefasciatus* (Malaysia) laboratory colony was obtained from Bayer (Bayer, Leverkusen, Germany). Both colonies were reared and maintained at 28 ± 5 °C and 80% humidity, with a 12 h light/dark cycle and 10% fructose ad libitum. 

### 2.2. Virus Stocks and Titration

A 70% confluent C6/36 cell flask (T75) was inoculated with ASALV [30]. Three days after inoculation, the cleared supernatant was collected and stored at −80 °C. ASALV titrations were performed using the TCID50 assay with C6/36 cells. 4 × 10^4^ cells/well were seeded in a 96-well plate with a cell medium (Leibovitz’s L15 medium Thermo Fisher Scientific, Inc., Waltham, MA, USA) supplemented with 10% foetal calf Serum (Thermo Fisher Scientific Inc., Waltham, MA USA) 1x penicillin/streptomycin (Thermo Fisher Scientific Inc., Waltham, MA, USA) and 10% tryptose phosphate broth (Gibco Life Technologies, Paisley, UK). Four replicates were used for each dilution. After three days of incubation at 28 °C, the cells were first checked for morphological changes, as low ISV titers can cause morphological changes without causing a clear CPE. Then, the cells were fixed with 8% formaldehyde and stained with crystal violet. The titer was calculated according to the Spearman–Kaeber algorithm [33].

### 2.3. ASALV Transmission Experiments

#### 2.3.1. Adult Infections through Feeding and Intrathoracic Injections

Between 7 and 13 days old female *Ae. aegypti* mosquitoes were either infected by intrathoracic injection or oral feeding. For intrathoracic injection, the mosquitoes were immobilised with CO_2_, kept on ice, and injected with ASALV (10^3^ PFU/mosquito, using Nanoject II (Drummond). For infections through feeding, mosquitoes were starved of fructose for 24 h and then fed with an ASALV-PBS solution (10^7^ PFU/mL, 10% ATP, and blue food dye) for 45 min, using the Hemotek membrane feeding system with synthetic chicken skin as the membrane. *Cx. quinquefasciatus* mosquitoes (Figure S1) were infected using the same virus titers and injection method, although, for oral infection, cotton buds were used to deliver the ASALV-PBS solution. Mosquitoes were anaesthetised with CO_2_, and fed mosquitoes were incubated at 28 ± 5 °C and 80% humidity, with a 12 h light/dark cycle and 10% fructose ad libitum. 

Inoculated mosquitoes were either harvested at 0, 7, or 14 days post-infection (dpi) or used in further experiments (salivation, dissemination, horizontal transmission assays).

#### 2.3.2. ASALV Dissemination

ASALV-fed or injected adult females were anaesthetised with CO_2_ and dissected at 7 and 14 dpi. The heads (including salivary glands), guts, ovaries, and carcasses of 15 mosquitoes were pooled per sample and homogenised in GMEM (Thermo Fisher Scientific, Inc., Waltham, MA, USA). The total RNA was isolated using Trizol LS (Invitrogen, Carlsbad CA, USA), according to the manufacturer’s protocol. To further investigate the dissemination in the head parts, heads, salivary glands and carcasses from ASALV-injected mosquitoes were dissected. Before homogenisation, the antenna and mouth parts were removed from the heads to investigate ASALV dissemination to the brain. The body parts of 5 mosquitoes were pooled per sample, and a total of 6 samples were processed. 

#### 2.3.3. ASALV Horizontal Transmission 

To investigate horizontal transmission during the adult life stage, ASALV-injected *Ae. aegypti* females were put in the same cage with uninfected male mosquitoes. A week later, males and females were collected and tested for ASALV infection.

To investigate the horizontal transmission during larval life stages, one-day-old mosquito larvae were transferred into a 96-well plate in a 30 µL larvae food solution (200 mL water + 0.2 g fish food pellet- Sera Wels Tabs XXL). 10^7^ PFU ASALV per well was added and incubated at 28° C ± 5 with a 12 h light/dark cycle. Two days post-infection, the larvae were transferred onto a 24-well plate in 1 mL food solution. A total of 300 µL of food solution was added every two days. After 6 days, the larvae were transferred to a clean 24-well plate, given fresh food, and left to pupate. The pupae were transferred to glass tubes, and emerging adults were collected and stored at −80 °C until analysed. The experiment was repeated twice independently. 

#### 2.3.4. Salivation Assay

A salivation assay was performed using ASALV-injected female mosquitoes 14 dpi. Mosquitoes were anaesthetised with CO_2,_ and their legs and wings were removed. They were forced to salivate in 10 µL of PBS in a cut filter tip, as previously described [32]. After 30 min of forced salivation, the tip containing the PBS-saliva mixture was removed and placed into a reaction tube containing 10 µL of PBS. Finally, 20 µL saliva + PBS was added to C6/36 seeded in 96 well plates. As no morphological changes or CPE was observed in the cells, to check whether viral RNA was present, the supernatant was collected from the saliva samples and some negative control cells. A QIAamp viral isolation kit (QIAGEN, Hilden, Germany) was used for RNA isolation from the supernatants. Corresponding female mosquito bodies were collected and homogenised in GMEM (Thermo Fisher Scientific, USA) without supplements, and RNA was isolated with Trizol LS (Invitrogen, Carlsbad CA, USA). ASALV quantification was performed as described below. 

#### 2.3.5. ASALV Vertical Transmission 

Between 7 and 10 days old female mosquitoes were either injected or fed with ASALV as described above. After a week, females were given a blood meal (50% blood, 10% Fetal Calf Serum, 1% ATP, completed with Fructose). Blood-fed females were transferred to a cage and left to lay eggs. The eggs were flooded after a drying period. L4 larvae and adults (females and males) were collected, homogenised, and isolated, while RNA was tested for ASALV presence by virus-specific RT-PCR. The experiment was repeated twice independently. 

### 2.4. ASALV Quantification

Whole mosquito samples or dissected tissue pools were homogenised in 500 µL or 250 µL of GMEM (Thermo Fisher Scientific, Waltham, MA, USA) without supplements, respectively. The homogenate was clarified by centrifuging, and 200 µL of the supernatant was used to extract RNA using TRIzol LS (Invitrogen, Carlsbad CA, USA) (3:1 /TRIzol: homogenate) according to the manufacturer’s protocol. The RNA concentration was measured using a nanodrop. RNA was either used directly (One-step quantitative (q)PCR), or cDNA was synthesised (to be used in qPCR) using 1 µg of RNA, random hexamers, and M-MLV reverse transcriptase (Promega, Fitchburg, WI, USA) according to the manufacturer’s protocol.

To quantify the ASALV load in different tissues, we performed two-step qPCR using cDNA samples and a QuantiTect SYBR Green PCR kit (QIAGEN, Hilden, Germany), according to the manufacturer’s protocol. The ASALV load was quantified relative to ribosomal S7 gene (housekeeper gene, *Ae. aegypti*) or GAPDH (housekeeper gene, *Cx. quinquefasciatus*, Figure S1) using previously published specific primers (ASALV, F: 5′-CCGTACTCGAAACAGACATTGC-3′, R: 5′-TCGTCAACGCCTAGATCCTCTA-3′; S7, F: 5′-CCAGGCTATCCTGGAGTTG-3′, R: 5′-GACGTGCTTGCCGGAGAAC-3′; GAPDH, F:5′-TCAAGCAGAAGGTCAAGGAAG-3′, R:5′-GTTGTCGTACCAGGAGATGAG-3′) [15,34,35]. A one-step qRT-PCR (QuantiTect SYBR Green RT-PCR Kit, QIAGEN, Hilden, Germany) was performed, according to the manufacturer’s protocol, to detect ASALV infection (infection rate, salivation assay, and horizontal transmission assays). Cp > 33.5 was considered negative based on the standard curve. 

All PCR reactions were performed in three technical replicates. The data were analysed using a LightCycler 480 II Instrument (Roche) and the LightCycler 480 software (version 1.5.0 SP4), either using absolute quantification or advanced relative quantification. Standard curves for each gene were created using dilutions of the specific PCR products, and genome copy numbers were calculated and used for both absolute and relative quantification assays. 

### 2.5. Polymerase Chain Reaction (PCR) 

For ASALV detection, the above-mentioned ASALV-specific forward and reverse primers were used in combination with cDNA as a template. The amplification was performed using Go Taq polymerase (Promega, Fitchburg, WI, USA) with deoxynucleoside triphosphates (dNTPs) and a 5x Green Go Taq buffer, with a final reaction volume of 50 μL. Thermocycling conditions for the first round of amplification were 95 °C for 2 min, followed by 32 cycles of denaturation (95 °C for 30 s), annealing (56 °C for 30 s), extension (72 °C for 45 s), and a final extension at 72 °C for 7 min. The amplification product was visualised in a 2% agarose-1× TAE (Tris-acetate-ethylenediaminetetraacetic acid) gel by ethidium bromide staining and UV transillumination. The expected size for amplification (PCR product) was 97 bp.

### 2.6. Small RNA Sequencing

The midguts from 15 mosquitoes (ASALV fed, 7 dpi) were pooled and homogenised in GMEM (Thermo Fisher Scientific, Waltham, MA, USA). The total RNA was isolated using TRIzol LS (Invitrogen, Carlsbad CA, USA) according to the manufacturer’s protocol, with glycogen as a carrier. For small RNA sequencing, libraries were prepared with 100 ng of the total RNA and the Nextflex small RNA-Seq kit v3 (PerkinElmer Inc., Waltham, MA, USA), and libraries with a size corresponding to (18–35 nucleotides after library preparation) were sequenced on a NovaSeq6000 SP v1.0 platform (2 × 50 bp) [29]. Sequencing was performed at IKMB (Kiel, Germany). The data were analysed, as described previously [23]. As a template, the ASALV genome was used (GenBank accession number MK959115). 

### 2.7. Data Analyses 

Data analyses were conducted in R version 4.1.2 (1 November 2021). To analyse the ASALV replication in whole mosquitoes at 0, 7 and 14 dpi, we used a non-parametric Kruskal–Wallis test. When there was a significant effect during the day, pairwise comparisons were made using a dunnTest (FSA package) with Bonferroni correction. To analyse the effects of *tissue type*, *dpi* (7, 14), and *treatment* (Injected, Fed) on the ASALV load in different tissues, we first fitted a generalised linear model from the Gaussian family using log-transformed data. Likelihood ratio tests of the full model against the model without a given effect were used to obtain the degrees of freedom, deviance and *p*-values. The statistical difference between the salivary gland, carcasses and brain tissues in terms of the ASALV load was tested using a pairwise-*t*-test with Bonferroni correction for multiple testing. A *p*-value < 0.05 was considered statistically significant.

## 3. Results

### 3.1. ASALV Infects and Replicates in Ae. aegypti 

**Figure 1:**
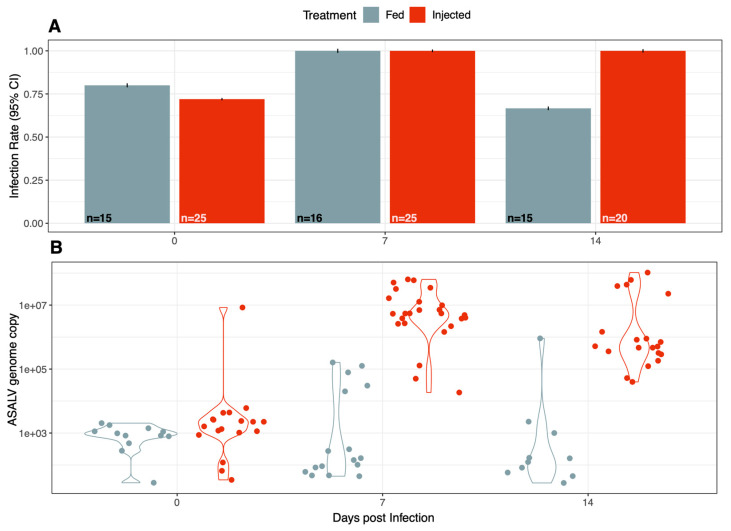
ASALV infection rate (**A**) and load (**B**) in female *Ae aegypti* mosquitoes following intrathoracic injection and oral infection. Adult female mosquitoes (7–13 days old) were fed or injected with ASALV and RNA was isolated from the collected mosquitoes at 0, 7 and 14 dpi. ASALV was quantified using the qRT-PCR infection rate (**A**), or genome copy numbers (**B**) were calculated using corresponding standard curves. Each data point represents individual mosquitoes (whole bodies). Error bars indicate 95% confidence intervals for the infection rate, n = sample size.

To determine whether ASALV could infect and replicate in *Ae. aegypti* mosquitoes, mosquitoes were either injected or fed with ASALV and were collected at different times post-infection (0, 7, and 14 dpi). ASALV was quantified by qRT-PCR. The samples from 0 dpi were ASALV-positive, confirming that both the feeding and injection experiments worked well. The injected mosquitoes showed a 100% infection rate on 7 and 14 dpi, while mosquitoes orally exposed to ASALV showed a lower infection rate on 14 dpi (Figure 1A). 

A general increase in the ASALV RNA copy number was observed at 7 (mean (±se), 10^5.17(±0.33)^) and 14 dpi (mean (±se), 10^4.89 (±0.37)^) compared to that at 0 dpi (mean (±se), 10^3.10(±0.17)^), suggesting the active infection and replication of ASALV in *Ae. aegypti* mosquitoes for both groups (Figure 1B). This increase was particularly high in the injected group with a 3 log increase from 0 dpi 10^3.26(±0.261)^ to 7 dpi 10^6.65(± 0.17)^(Kruskal–Wallis χ^2^ = 44, df = 2, *p* < 0.001; Funn test: 0–7 dpi *p* < 0.001; 0–14 dpi *p* < 0.001). In the fed group, the ASALV load did not differ significantly between 0, 7 and 14 dpi (Kruskal–Wallis χ^2^ = 4, df = 2, *p* > 0.05), and only some samples collected at 7 dpi and 14 dpi exhibited a higher ASALV load compared to 0 dpi (Figure 1B). This suggested a strong bottleneck passing the midgut barrier when the mosquitoes were fed with ASALV. Nevertheless, many mosquitoes remained positive at 14 dpi, indicating that ASALV could infect and replicate in *Ae. aegypti* by feeding.

### 3.2. ASALV Interacts with the RNAi Response in the Midgut 

**Figure 2:**
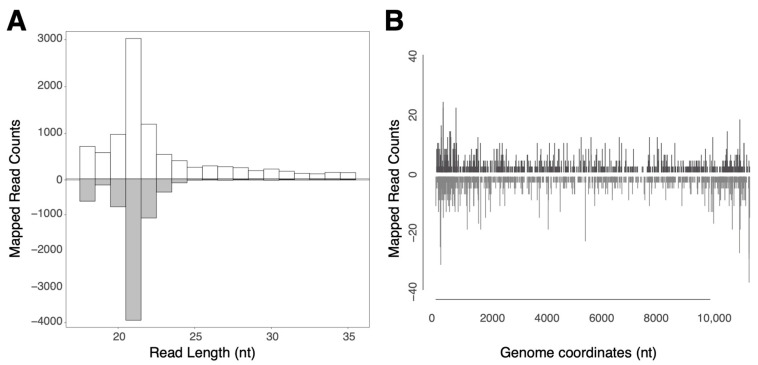
ASALV-specific small RNA production in the *Ae. aegypti* female midgut. Adult female *Ae. aegypti* mosquitoes were fed with ASALV, and the midgut of 15 female mosquitoes was pooled at 14 dpi. Size distribution of small RNAs mapping to the ASALV genome (white) or antigenome (grey) (**A**). Distribution of the 21 nt small RNAs along the ASALV genome (white) and antigenome (grey) (**B**).

The total RNA of the dissected midgut pool of 15 ASALV-injected mosquitoes was sequenced and analysed to investigate ASALV-specific small RNA production. ASALV-specific 21 nt-sized siRNAs were produced in the midgut (Figure 2A). These vsiRNAs were produced along the ASALV genome and antigenome for a similar amount (Figure 2B). We observed ASALV-specific piRNA-sized small RNAs (mostly mapping to the genome) in the midgut. However, these did not show ping-pong amplification signals (data not shown).

### 3.3. ASALV Disseminates to Different Tissues and Replicates Mostly in Heads 

**Figure 3:**
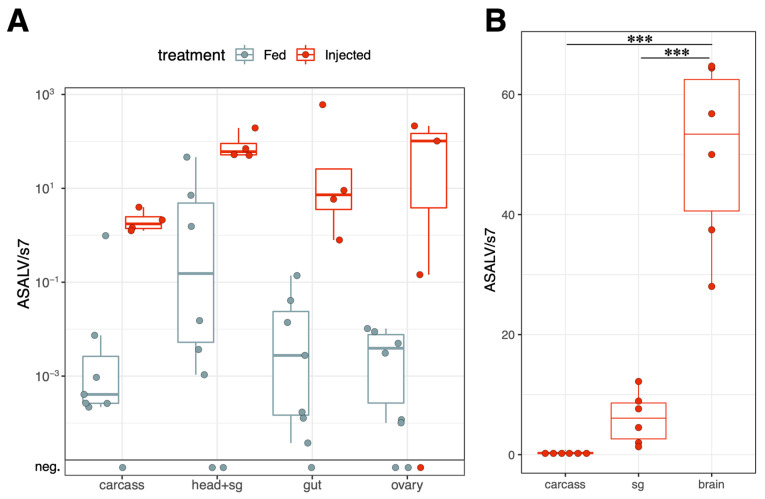
ASALV dissemination to different tissues in ASALV-fed or injected adult *Ae. aegypti* females. The RNA of different tissue pools (15 mosquitoes/pool), dissected at 7 and 14 dpi, was isolated and the ASALV load was determined by qPCR (**A**). RNA of brain tissue, salivary glands (sg) and carcass pools (5 mosquitoes/pool) were isolated from ASALV-injected mosquitoes at 7 dpi (**B**). Six pools per tissue were analysed for ASALV load in the carcass, salivary gland and brain in ASALV-injected *Ae. aegypti* females. ASALV load was determined by qPCR and relative ASALV quantification using the ribosomal S7 gene as a housekeeping gene. ***: *p* < 0.001. Each data point represents dissected tissue pools consisting of 15 (**A**) or 5 (**B**) mosquitoes.

The ASALV load was quantified using qPCR in pooled tissue samples from intrathoracically injected and orally infected mosquitoes. In both groups, ASALV was disseminated successfully to all dissected tissues (Figure 3A). Although we performed the dissections at 7 and 14 dpi separately, there was no difference between 7 and 14 dpi samples in terms of the ASALV load, so these data were pooled together in Figure 3A (glm, df = 1, dev = 3.27, *p* = 0.15). A major difference was caused by the infection route; injected mosquitoes generally had higher ASALV genomes compared to fed mosquitoes (glm, df = 1, dev = 109, *p* < 0.001; Figure 3A). There was no significant effect in the interaction of the infection route and tissue type (glm, df = 3, dev = 2.64, *p* > 0.05), showing that the injection route did not change the tissue tropism of ASALV. Viral load differed between dissected tissues (glm, df = 3, dev = 18.6, *p* < 0.001), with the highest ASALV load observed in head pools, including the salivary glands (Figure 3A). To determine whether the tissue tropism of ASALV was specific to *Ae. aegypti,* or if it exhibited the same tissue tropism in another mosquito species, we checked the ASALV tissue tropism in *Cx. quinquefasciatus* using the same infection protocols as *Ae. aegypti*. No specific tropism for the head was observed in *Cx. quinquefasciatus* female mosquitoes (Figure S1). 

To investigate which tissue was responsible for the high ASALV load in the heads, the experiment was repeated. This time, in addition to the carcass, the heads of ASALV-injected mosquitoes (at 7 dpi) were separated to create pools of the salivary glands and brains (Figure 3B). ASALV load was highest in the brain pool compared to the salivary glands (pairwise-*t* test with Bonferroni correction, *p* < 0.001) and carcasses (pairwise-t test with Bonferroni correction, *p* < 0.001). ASALV load in the carcass was not significantly different from salivary glands.

### 3.4. ASALV Transmission Routes in Ae. aegypti

To understand how ASALV might be transmitted in nature, we checked horizontal transmission during adult and larval stages. Since ASALV was detected in the salivary glands, its transmission through saliva was checked. For this, ASALV-injected *Ae. aegypti* was used to perform a forced salivation assay at 14 dpi. We did not observe any infection signs in the saliva-incubated mosquito cells, nor was ASALV RNA detected in the collected cell supernatants. However, all salivated females were positive. 

Due to the presence of ASALV in the ovaries, we investigated the vertical transmission of ASALV in F1 offspring. Therefore, ASALV’s presence in F1 Larvae (10 L4 tested per group) or adult pools (10 pools of 5 males or females tested per group) derived from ASALV-injected or -fed females was determined by RT-PCR. Experiments were repeated twice for each group, but no ASALV was detected in the F1 larvae or adults. 

**Figure 4:**
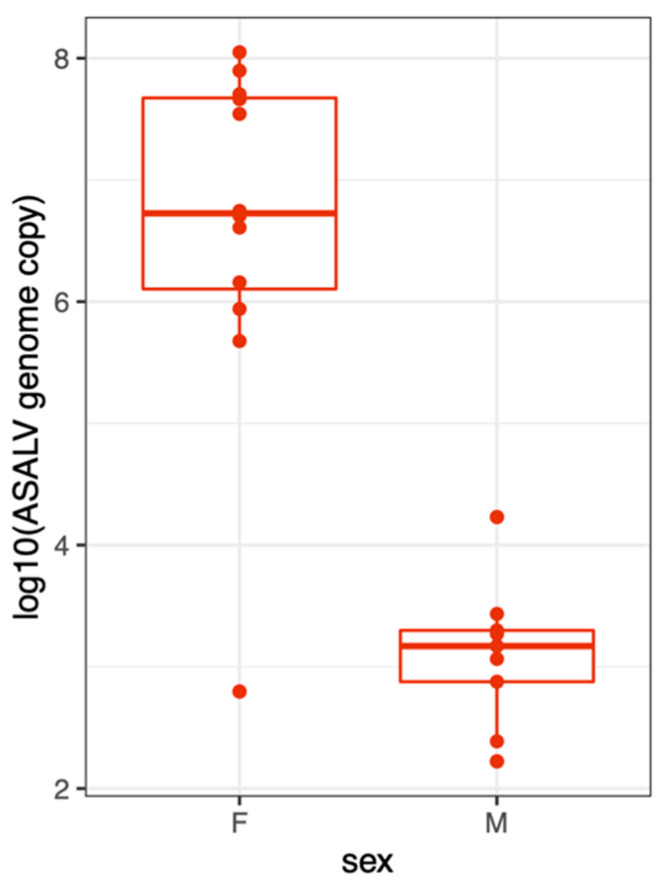
ASALV can be horizontally transmitted from female to male *Ae. aegypti* mosquitoes. ASALV-injected females were kept with uninfected males for 7 days. ASALV genome copy numbers were quantified in female and male mosquitoes using qRT-PCR. The results represent the combined data of two independent repeats. Each data point represents an individual mosquito (whole body sample).

To investigate the horizontal transmission between adult mosquitoes, we placed 5–7 females in a cage with 15 uninfected males in two independent replicates (12 females and 30 males in total). A week later, surviving females (n = 12) and males (n = 18) were collected, and the ASALV load was quantified using qRT-PCR. All tested females were highly infected with ASALV (Figure 4). Out of the 18 males tested, 9 were infected, indicating a 50% transmission rate (95% CI: 26-73%). However, ASALV load was lower in the males (mean(±se):10^3(±0.2)^) compared to the females (mean(±se):10^6.62(±0.42)^) that were injected.

To investigate the horizontal transmission during larval stages, mosquito larvae were reared in water harbouring ASALV. Emerging adult mosquitoes were collected and tested for the presence of ASALV by PCR. A total of 10% (95% CI: 2–20%) of the tested adult mosquitoes (n = 29, in three independent experiments) were ASALV-positive, suggesting the possibility for ASALV horizontal transmission during larval stages.

## 4. Discussion

ISVs can only replicate in insects and insect-derived cell lines and not in vertebrates. A large number of ISVs have been discovered as a result of recent advances in sequencing technology and increased field mosquito surveillance. However, our knowledge of ISV host range, their effects on mosquito fitness, and how they are maintained and transmitted in nature is limited. To address some of these open questions, we studied the replication kinetics, dissemination, and transmission of ASALV in vector mosquito species, *Ae. aegypti*.

Despite the increase in the detection of many diverse ISVs by sequencing studies, ISVs’ host range was not always clearly defined. In the case where the discovered ISV was isolated, its replication in mosquito cells and vertebrate cells was checked. However, the replication kinetics of ISVs are rarely checked in other insects, with only several exceptions [21,36]. ASALV cannot replicate in cell lines derived from ectothermic hosts of the Culex mosquito (frogs, snakes and fish-derived cell lines), suggesting that ASALV is an ISV [19]. Previous studies have demonstrated ASALV replication in cell lines derived from Aedes species, even though it was isolated from *Culex declarator* mosquitoes [19,23]. In this study, we showed that *Ae. aegypti* females are susceptible to ASALV when intrathoracically and orally infected. The infection rates in intrathoracically injected mosquitoes were higher than in orally infected mosquitoes, and the ASALV load in whole mosquitoes increased significantly in the ASALV-injected mosquitoes. On the other hand, ASALV load did not significantly increase in the whole mosquitoes following oral infection with ASALV, but mosquitoes stayed positive at 14 dpi, supporting its active replication. In addition, the presence of ASALV-specific 21-nt vsiRNAs in *Ae. aegypti* midgut showed that ASALV infected and replicated in the midgut following oral infection, which triggered the host RNAi response. In addition to vsiRNAs, piRNA-sized ASALV-specific small RNAs were also detected; these lacked ping-pong amplification signals, similar to previous findings in *Ae. albopictus*-derived (U4.4) cells [19] and *Ae. aegypti*-derived cells (RNAi-competent AF5 cells) [23]. Overall, our results show that ASALV infects and replicates in *Ae. aegypti*, similar to the results of previous studies showing successful EILV infection in *Ae. aegypti* and *Cx. tarsalis*. [20,21]. To date, all insect-specific alphaviruses have been discovered in Culex or Anopheles species, although the successful replication of EILV and ASALV in *Ae. aegypti* mosquitoes [15,16,17,18,19,20,21] suggest that *Ae. aegypti* could be a potential host for insect-specific alphaviruses.

When a virus is orally acquired, the midgut is the first organ in the mosquito to become infected. Following midgut infection, the virus spreads to secondary tissues, such as the salivary glands [37,38]. Previous research has shown that when infected orally, *Ae. aegypti, Anopheles gambiae*, and *Cx. quinquefasciatus* mosquitoes experienced low dissemination of EILV to their legs and wings. When infected orally, a high infectious titer was necessary for dissemination in *Ae. aegypti* (10^7^ PFU/mL), *Anopheles gambiae* (10^7^ PFU/mL), and *Cx. quinquefasciatus* (10^9^ PFU/mL) compared to the injected ones [20]. This suggests a strong midgut barrier against insect-specific alphaviruses. In this study, ASALV was disseminated successfully in *Ae. aegypti* with a viral titer of 10^7^ PFU/mL, similar to EILV. However, since we did not determine the dissemination rate (i.e., by testing individual mosquitoes) in this study, our results for ASALV cannot be directly compared to the previously reported dissemination rates of EILV with the same titer. We also observed a significant impact in terms of the route of infection on the ASALV load in dissected tissues: ASALV-injected mosquitoes had higher viral loads than orally infected mosquitoes. Nevertheless, ASALV successfully disseminated in different tissues in both ASALV-injected and orally infected mosquitoes, and the infection route did not change the tissue tropism of ASALV in *Ae. aegypti*.

Another open question on ISVs is how they affect mosquitos’ fitness. Some ISVs with DNA genomes (e.g., Aedes albopictus densoviruses) exhibited visible pathology and mass mortality during the larval stage [39], whereas insect-specific RNA viruses did not seem to cause any easily detectable pathology or high mortality. However, some RNA viruses have been shown to affect mosquito behaviour. For instance, CxFV infection alters the flight activity of naturally infected *Cx. pipiens,* which can, in return, interfere with the host-seeking behaviour of the mosquito, resulting in reduced fitness [40]. Similarly, the arthropod-borne dengue virus caused a higher level of locomotor activity in infected *Ae. aegypti* compared to uninfected mosquitoes [41]. Here, interestingly, we observed a high level of ASALV in the brain, suggesting a tissue tropism for ASALV. It would be interesting to see if the presence of ASALV in the brain of a mosquito affected its behaviour. Further studies are required to understand the effect of ISVs infection on mosquito behaviour and fitness.

ISVs can be transmitted via various transmission (horizontal and vertical) routes during different mosquito life stages. These differences in the transmission route can have important roles in the ecology of these viruses [3,42]. Here, we studied the various possible transmission routes of ASALV, including horizontal transmission routes that can occur during adult and larval stages and vertical transmission. Our results showed that ASALV transmitted horizontally during both adult (from intrathoracically infected females to males) and larval stages. This suggested that ASALV could be horizontally transmitted in nature. However, it should be kept in mind that we did not test for different environmental conditions, which could affect viral infectivity (UV, temperature variance, pH) during larval stages, and used an artificial infection route (intrathoracic injection) for adult-stage infection. Hence, the efficiency of these transmission routes could differ in natural conditions. While we detected ASALV RNA in the salivary glands and ovaries, no infectious ASALV particles were present in mosquito saliva, and the F1 generation of ASALV-injected or fed females was ASALV negative, too. A recent study showed that EILV is not vertically transmitted, but it has been found in saliva in *Cx. tarsalis* [21]. The absence of infectious ASALV in infected females’ saliva ruled out transmission via expectorated saliva. These findings might also indicate that female-to-male transmission is mainly venereal. While we focused on female mosquitoes in the tested adult stages due to their importance in arbovirus transmission and, thus, arbovirus control, it is also possible that male mosquitoes are a significant contributor to ASALV transmission in nature. 

The transmission routes could define how widespread, geographically or in terms of host range, certain ISVs are found. For instance, several insect-specific flaviviruses can be transmitted vertically [6,21,43,44] and some horizontally [45], although vertical transmission has not been reported for alphaviruses, and this could be one of the explanations why only a small number of insect-specific alphaviruses have been found in contrast to flaviviruses with worldwide distributions [46].

ISVs have attracted significant scientific attention in the last decade as some of them can interfere with arbovirus replication in mosquitoes and, thus, can be used as an arbovirus control tool. Arbovirus interference has been shown for insect-specific alphavirus, EILV, although ASALV–arbovirus interactions have not yet been studied [22]. While ISV research in terms of their interactions with arboviruses is rapidly growing, there are still many open questions about how they are dispersed in nature and how they affect their hosts. The knowledge of ASALV’s tissue tropism provides important information for further studies on the effects of ISVs on their hosts (e.g., behavioural changes and related fitness costs), which could lead to novel applications of ISVs as vector controls. On the other hand, transmission routes are essential to understand for their potential use as an arbovirus or vector control, especially to devise release strategies.

## 5. Conclusions

We demonstrated that ASALV (i) infects *Ae. aegypti*, when infected orally and intrathoracically, (ii) is transmitted horizontally but not vertically, (iii) disseminates to different tissues but shows a tropism toward the brain, and (iv) induces a host RNAi response in the midgut. Our results provide a detailed picture of the dissemination, infection and transmission routes of ASALV in *Ae. aegypti*, which could ultimately help develop novel arbovirus or vector control strategies involving ISVs or serve as a model system to study ISV and mosquito–arbovirus interactions. 

## Data Availability

Small RNA sequencing data are available in the NCBI Sequence Read Archive under BioProject ID PRJNA957818. The RT-PCR and qRT-PCR data will be made available upon request.

## Supplementary Materials

The following supporting information can be downloaded at: https://www.mdpi.com/article/10.3390/v15051113/s1, Figure S1: ASALV dissemination in *Cx. quinquefasciatus.*
