# Supplementary material for: Agua Salud Alphavirus Infection, Dissemination and Transmission in Aedes aegypti Mosquitoes
Source: Viruses. 2023 May 3;15(5):1113. doi: 10.3390/v15051113 (PMC10223791; doi:10.3390/v15051113)
Supplement: Supplementary file 1 [file viruses-15-01113-s001.zip › viruses-2331620-supplementary.pdf]

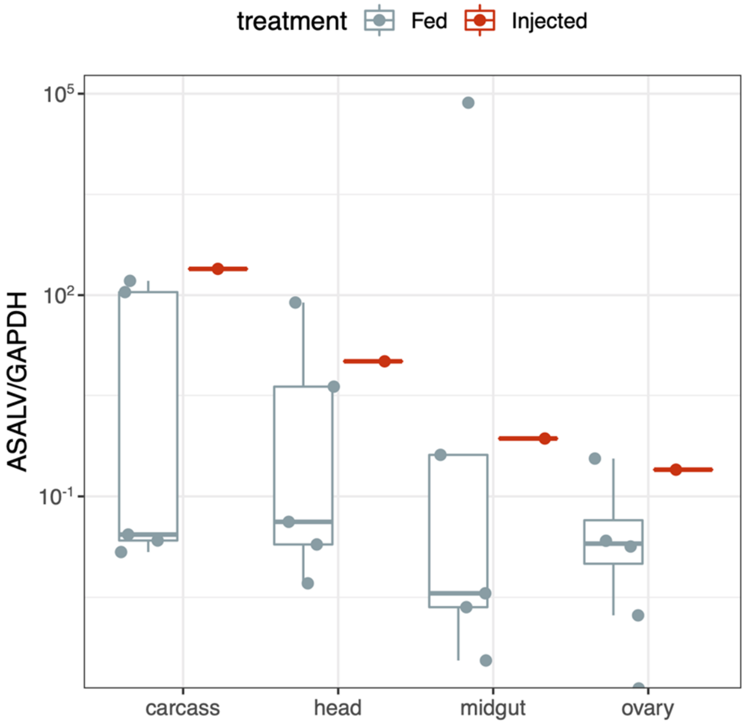

Figure S1. ASALV dissemination in *Cx. quinquefasciatus*. 7-13 days old female *Cx. quinquefasciatus* fed or injected with ASALV were dissected at 7 and 14 dpi to check whether there was a tissue tropism to head as seen for *Ae. aegypti*. Infections were performed using the same viral titer with *Ae. aegytpi* experiments. The heads (including salivary glands), guts, ovaries, and carcasses of 15 mosquitoes were pooled and homogenised in GMEM. The total RNA of tissue pools was isolated, and the relative ASALV load was determined by qPCR GAPDH as a housekeeper. Each data point represents dissected tissue pools of 15 mosquitoes.
